# The analysis of serum lipids profile in Guillain-Barre syndrome

**DOI:** 10.3389/fimmu.2023.1301577

**Published:** 2023-12-08

**Authors:** Lijuan Wang, Yaowei Ding, Jie Liu, Guanghui Zheng, Siwen Li, Wencan Jiang, Kelin Chen, Xin Luan, Yuxin Chen, Siqi Wang, Guojun Zhang

**Affiliations:** ^1^ Department of Clinical Diagnosis, Laboratory of Beijing Tiantan Hospital, Capital Medical University, Beijing, China; ^2^ Beijing Engineering Research Center of Immunological Reagents Clinical Research, Beijing Tiantan Hospital, Capital Medical University, Beijing, China; ^3^ National Medical Products Administration (NMPA) Key Laboratory for Quality Control of In Vitro Diagnostics, Beijing Tiantan Hospital, Capital Medical University, Beijing, China

**Keywords:** Guillain-Barre syndrome, lipid, immune status, inflammatory markers, infection

## Abstract

**Background:**

Guillain-Barre syndrome (GBS) is an immune-mediated inflammatory peripheral neuropathy. This study aimed to conduct a systematic analysis of the serum lipids profile in GBS.

**Methods:**

We measured the serum lipids profile in 85 GBS patients and compared it with that of 85 healthy controls matched for age and sex. Additionally, we analyzed the correlation between lipids and the severity, subtypes, precursor infections, clinical outcomes, clinical symptoms, immunotherapy, and other laboratory markers of GBS.

**Results:**

Compared to the healthy controls, GBS exhibited significantly elevated levels of Apolipoprotein B (APOB), Apolipoprotein C2 (APOC2), Apolipoprotein C3 (APOC3), Apolipoprotein E (APOE), triglycerides (TG), and residual cholesterol (RC). Conversely, Apolipoprotein A1 (APOA1), Apolipoprotein A2 (APOA2), and high-density lipoprotein (HDL) were substantially lower in GBS. Severe GBS displayed noticeably higher levels of APOC3 and total cholesterol (TC) compared to those with mild disease. Regarding different clinical outcomes, readmitted GBS demonstrated higher RC expression than those who were not readmitted. Moreover, GBS who tested positive for neuro-virus antibody IGG in cerebrospinal fluid (CSF) exhibited heightened expression of APOC3 in comparison to those who tested negative. GBS with cranial nerve damage showed significantly reduced expression of HDL and APOA1 than those without such damage. Additionally, GBS experiencing limb pain demonstrated markedly decreased HDL expression. Patients showed a significant reduction in TC after intravenous immunoglobulin therapy. We observed a significant positive correlation between lipids and inflammatory markers, including TNF-α, IL-1β, erythrocyte sedimentation rate (ESR), white blood cells, monocytes, and neutrophils in GBS. Notably, APOA1 exhibited a negative correlation with ESR. Furthermore, our findings suggest a potential association between lipids and the immune status of GBS.

**Conclusion:**

The research demonstrated a strong connection between lipids and the severity, subtypes, clinical outcomes, precursor infections, clinical symptoms, immunotherapy, inflammation, and immune status of GBS. This implies that a low-fat diet or the use of lipid-lowering medications may potentially serve as an approach for managing GBS, offering a fresh viewpoint for clinical treatment of this condition.

## Background

Guillain-Barre syndrome (GBS) is a prevalent disorder characterized by demyelination of both spinal and peripheral nerves. It affects an estimated 1-2 individuals per 100,000 annually worldwide (1, 2) and stands as the leading cause of global acute flaccid paralysis (3). The initial symptom typically manifests as weakness in the lower limbs, peaking within a span of 3-15 days. More than 90% of patients cease progression within 4 weeks and achieve complete recovery within weeks or months. However, 1.7%-5% of patients encounter life-threatening tetraplegia and respiratory paralysis, while 10%-15% experience long-term complications, such as muscle weakness in both the lower and upper limbs, muscle atrophy, muscle pain, and foot drop (4). Certain patients may also present with facial palsy, dysphagia, dysarthria, choking, and sputum retention. Also, vegetative dysfunction in some individuals manifests as either excessive or insufficient sweating in the hands and feet, dry skin on the extremities, or urinary and fecal retention or incontinence.

Given the ongoing improvement in quality of life, the prevalence of hyperlipidemia has reached unprecedented levels. This condition significantly impacts the physical and mental well-being of individuals, raising substantial societal concerns. Recent studies have highlighted a potential correlation between abnormal serum lipids profile and GBS.

In our previous study investigating proteomic differences between the cerebrospinal fluid (CSF) samples of GBS patients and the control group, we observed that the identified proteins were primarily enriched in pathways associated with lipid metabolism (5). Additionally, our study revealed significantly higher expression of APOC3 in the CSF and serum of GBS patients compared to other neurological disease groups and healthy individuals undergoing physical examination (5). A recent investigation demonstrated that APOC3 induces the activation of the NLRP3 through Caspase8 and TLR2/4, leading to the release of interleukins and amplification of the inflammatory response (6). Correspondingly, animal experiments demonstrated that APOC3 exacerbates tissue damage and delays tissue repair. Several studies have reported reduced levels of APOE in the CSF of GBS patients, and it has been suggested that blood-brain barrier dysfunction, caused by APOE deficiency, may increase susceptibility to GBS and worsen the clinical symptoms of affected individuals (7). APOE has been shown to suppress the expression, augmentation, and activation of CD4^+^ T cells, regulate the Th1/Th2 balance, suppress Th1 and Th17 cells, and participate in the immune response in experimental autoimmune neuritis (EAN), an animal model of GBS (8, 9). APOA1, the primary protein component of HDL, is a multifunctional protein involved in cholesterol transport, inflammation regulation, and immune response. It inhibits the production of pro-inflammatory cytokines by activated T cells. APOA1 can also bind and neutralize lipopolysaccharide (LPS), a Gram-negative product, leading to reduced activation of TLR4 and decreased release of TNF-α and IL-1β, thereby alleviating clinical symptoms in GBS patients (10). In recent years, the concept of cholesterol toxicity has emerged, suggesting that hypercholesterolemia may compromise the integrity of the blood-brain barrier (11, 12). Statins are commonly used lipid-lowering medications, and several studies have demonstrated their effective therapeutic effects in animal models of GBS (13). Apolipoprotein D (APOD) is a small glycoprotein responsible for the localized transport of small hydrophobic ligands. APOD exhibited high expression in GBS and showed a correlation with increased levels of proteins in the CSF and a compromised blood-nerve barrier (14).

To date, there is a lack of large-scale clinical trials that comprehensively investigate the relationship between lipids and GBS, and the existing evidence is controversial. Therefore, we conducted a prospective analysis of 13 lipid levels and retrospectively collected clinical data from a substantial cohort of GBS patients. Our objective was to thoroughly analyze serum lipids profile in GBS and explore the correlation of lipids with the severity, prognosis, pre-infection status, clinical symptoms, and other laboratory parameters of GBS.

## Methods

### Patients and healthy controls

A total of 85 patients diagnosed with GBS based on the diagnostic criteria (15) were selected as the GBS group from December 2019 to April 2022 at Beijing Tiantan Hospital of Capital Medical University. The study also enrolled 85 healthy controls whose gender and age were not statistically different from the GBS group. All healthy controls had no history of infection, autoimmune diseases, or other medical conditions. We measured the serum lipids profile in GBS and healthy controls. Also, we analyzed the correlation between lipids and the clinical data and other laboratory markers of GBS.

### Data collection

Clinical data were retrospectively collected, including information on antecedent infections, cranial nerve damage, limb pain, Hughes scores at admission, nadir, and discharge. Blood samples were collected upon admission from all GBS patients. Peripheral whole blood was utilized for the analysis of various parameters including white blood cell count (WBC), neutrophils (NEUT), monocytes (MONO), erythrocyte sedimentation rate (ESR), and lymphocyte subsets such as CD19^+^ cells (B cells), CD3^+^ T cells (ToT), CD4^+^ T cells (Th), CD8^+^ T cells (Ts), and the CD4^+^/CD8^+^ ratio (Th/Ts). In addition, serum samples were employed to measure levels of APOA1, APOA2, APOB, APOC2, APOC3, APOE, TG, TC, HDL, low-density lipoprotein (LDL), oxidized-LDL (ox-LDL), lipoprotein a (LPa), TNF-α, IL-1β, and immunoglobulin G (IgG). Remnant cholesterol (RC) was calculated by subtracting HDL and LDL from TC. CSF samples were obtained through lumbar puncture, performed 2-3 weeks after disease onset. These CSF samples were analyzed for IgG levels and the presence of neurological virus antibodies such as Cytomegalovirus (CMV), Toxoplasmavirus (TOX), Rubella virus (RUB), Herpes simplex virus (HSV), and Epstein-Barr virus (EBV). CSF samples with one or more positive results were categorized as CSF neurovirus IgG (+), while the negative group was labeled as CSF neurovirus IgG (-).

### Evaluation of disease severity and short-term prognosis

The clinical scores of the 85 GBS patients were assessed at three time points (admission, nadir, and discharge) using the widely used Hughes Functional Grading Scale (HFGS), a disability assessment tool specifically designed for GBS patients (16). The grading criteria were as follows: 0, no symptoms; 1, mild symptoms with the ability to run; 2, ability to walk independently for 10 meters or more, but unable to run; 3, ability to walk 10 meters with assistance; 4, bedridden or wheelchair-bound; 5, requiring supplementary ventilation for at least part of the day; 6, deceased.

A GBS disability score of ≥3 at admission or at nadir was considered as severe GBS, whereas a GBS disability score of < 3 at admission or at nadir was labeled as mild GBS. Poor short-term prognosis was defined as a GBS disability score of ≥3,while good short-term prognosis was indicated by a GBS disability score of < 3 at discharge.

### Statistical analysis

Statistical analysis was performed using SPSS software (version 20.0). Normally distributed continuous variables were compared using the Student’s t-test, whereas non-normally distributed data were assessed using the Mann-Whitney U test. The Spearman rank correlation coefficient was employed to analyze the relationship between clinical characteristics and serum profile. A p-value less than 0.05 was considered statistically significant (*p<0.05, **p<0.01, ***p<0.001).

## Results

### Comparison of serum lipids profile in GBS and healthy controls

**Figure 1:**
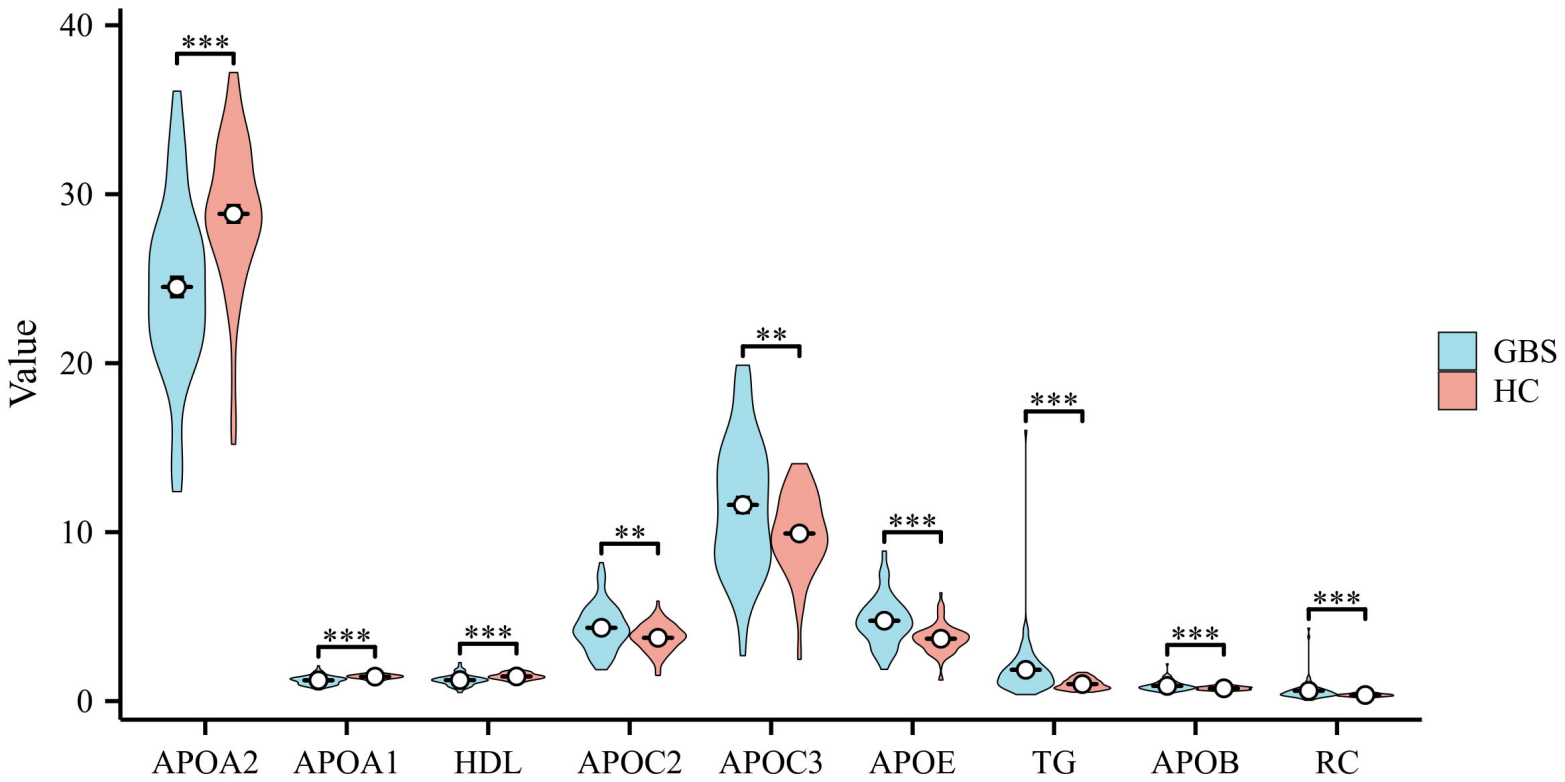
Comparison of Serum Lipids Profile in GBS and Healthy Controls Differences in the expression levels of Apolipoprotein B (APOB), Apolipoprotein C2 (APOC2), Apolipoprotein C3 (APOC3), Apolipoprotein E (APOE), triglycerides (TG), and residual cholesterol (RC) were observed, with noticeably higher levels in Guillain-Barre syndrome (GBS) subjects than in healthy controls (HC). Conversely, Apolipoprotein A1 (APOA1), Apolipoprotein A2 (APOA2), and high-density lipoprotein (HDL) exhibited markedly lower levels in GBS patients compared to the HC. No statistically significant differences were found in total cholesterol (TC), low-density lipoprotein (LDL), oxidized-LDL (ox-LDL), lipoprotein a (LPa) between the two groups (**p<0.01, ***p<0.001).

There were a total of 85 patients with GBS and 85 healthy controls included in this investigation. The characteristics and 13 serum lipids of GBS and healthy controls are presented in **Table 1**, namely APOA1, APOA2, APOB, APOC2, APOC3, APOE, TG, TC, HDL, LDL, RC, oxLDL, and LPa. As depicted in **Figure 1**, APOB, APOC2, APOC3, APOE, TG, and RC exhibited significantly higher expression levels, while APOA1, APOA2, and HDL were markedly lower in GBS compared to healthy controls (HC). TC, LDL, oxLDL, and LPa showed no statistical differences between the two groups.

**Table 1:** Clinical and laboratory characteristics in patients with GBS and healthy controls.

Characteristics	Healthy Controls	Patients with GBS	P
n	85	85	
Sex,male, n (%)			1.000
Female	34 (20%)	34 (20%)	
Male	51 (30%)	51 (30%)	
Age, y	45 (36, 57)	52 (33, 63)	0.397
Hyperlipidemia	10 (5.9%)	51 (30%)	< 0.001
Diabetes	2 (1.2%)	23 (13.5%)	< 0.001
Hypertension	22 (12.9%)	30 (17.6%)	0.183
Heart disease	7 (4.1%)	8 (4.7%)	0.787
APOA2	29 (26.7, 31.7)	24.6 (21.3, 27.4)	< 0.001
APOC2	3.81 (3.29, 4.25)	4.19 (3.5, 5.23)	0.002
APOC3	9.99 (8.62, 11.78)	11.71 (8.7, 14.42)	0.007
APOE	3.73 (3.27, 4.07)	4.6 (3.9, 5.47)	< 0.001
TG	0.95 (0.75, 1.21)	1.53 (0.97, 2.12)	< 0.001
TC	4.42 (3.93, 4.74)	4.33 (3.79, 5.01)	0.820
HDL	1.47 (1.33, 1.61)	1.23 (1.0375, 1.3925)	< 0.001
LDL	2.52 (2.24, 2.87)	2.505 (1.975, 3.105)	0.995
APOA1	1.4499 ± 0.15161	1.2329 ± 0.266	< 0.001
APOB	0.77 (0.67, 0.84)	0.835 (0.72, 1.0225)	< 0.001
RC	0.36 (0.3, 0.42)	0.49 (0.35, 0.68)	< 0.001
Lpa	9.005 (4.9475, 24.552)	11.665 (6.8475, 20.07)	0.328
oxLDL	52.414 (22.346, 71.795)	42.169 (22.159, 56.655)	0.150

### Correlation of serum lipids with GBS severity and subtypes

**Figure 2:**
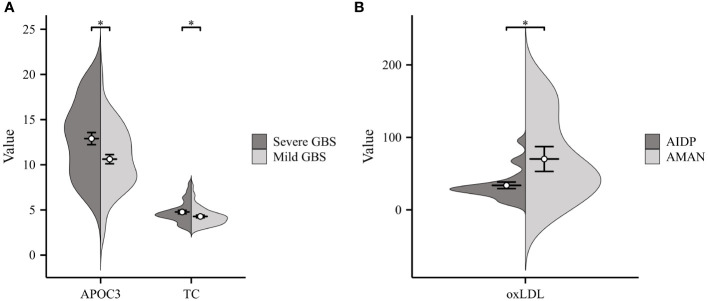
Correlation of Serum Lipids with GBS Severity and Subtypes. **(A)** Severe GBS displayed considerably higher levels of APOC3 and TC compared to those with Mild GBS. **(B)** Significantly higher levels of ox-LDL were observed in patients with acute motor axonal neuropathy (AMAN) subtype compared to those with acute inflammatory demyelinating polyneuropathy (AIDP) subtype (*p<0.05).

GBS can generally be categorized into four subtypes: acute inflammatory demyelinating polyneuropathy (AIDP), acute motor axonal neuropathy (AMAN), acute motor sensory axonal neuropathy (AMSAN), and Miller-Fisher syndrome (MFS) (17, 18). Due to the limited number of cases for AMSAN and MFS, our lipid analysis focused on comparing AMAN (n=11) and AIDP (n=21). As illustrated in **Figure 2B**, oxLDL exhibited significantly higher levels in patients with AMAN compared to those with AIDP. Additionally, according to the Hughes Functional Grading Scale (HFGS), GBS patients were classified as having either mild or severe disease. Notably, APOC3 and TC were considerably higher in patients with severe GBS (n=37) compared to those with mild disease (n=48) (as shown in **Figure 2A**).

### Relationship between serum lipids and infection in GBS

**Figure 3:**
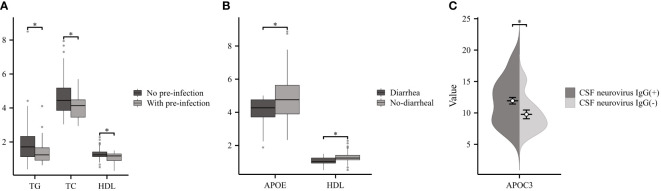
Relationship between Serum Lipids and Infection in GBS. **(A)** GBS with pre-infection demonstrated significantly lower levels of TG, TC, and HDL compared to No pre-infection group. **(B)** Patients with diarrhea had lower APOE and HDL than the No diarrhea. **(C)** Patients with cerebrospinal fluid (CSF) neurovirus IgG (+) exhibited a considerable increase in the expression of APOC3 compared to those with CSF neurovirus IgG (-) (*p<0.05).

Two-thirds of GBS patients reported experiencing precursor infections (pre-infection) prior to the onset of neurological symptoms (19). As illustrated in **Figure 3A**, a comparison was made between GBS patients with pre-infection (n=29) and No- pre-infection group (n=56). Patients with pre-infection displayed significantly lower levels of TG, TC, and HDL compared to the control group. The GBS patients were divided into two groups according to whether they had diarrhea before the onset of the disease, and the results showed that the diarrhea group (n=12) had lower APOE and HDL than the No diarrhea group (n=73) (**Figure 3B**). Additionally, a total of five neurological viruses, including CMV, TOX, RUB, HSV, and EBV, were selected for our study, and we detected CSF IgG antibodies. CSF neurovirus IgG (+) (n=50) referred to the presence of one or more positive results, while the absence of neurovirus IgG was denoted as CSF neurovirus IgG (-) (n=15). Our findings indicated a significant increase in APOC3 expression in patients with neurovirus IgG (+) compared to those with CSF neurovirus IgG (-) (**Figure 3C**).

### Serum lipids in GBS with different clinical outcomes

**Figure 4:**
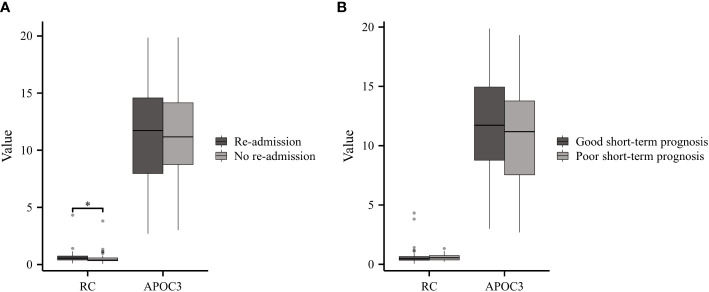
Serum Lipids in GBS with Different Clinical Outcomes. **(A)** Re-admitted GBS patients exhibited higher levels of RC compared to the No re-admitted group, while no significant difference in APOC3 expression was observed between the two groups. **(B)** There were no statistically meaningful differences in the levels of RC and APOC3 between patients with a good or poor short-term prognosis (*p<0.05).

GBS patients can exhibit varying clinical outcomes. Based on their readmission status, GBS patients were classified into two groups: Re-admitted (n=40) and No re-admitted (n=45). The results demonstrated higher levels of RC in re-admitted patients compared to the controls; however, no significant difference was observed in APOC3 expression between the two groups (**Figure 4A**). According to the Hughes Functional Grading Scale (HFGS), GBS patients were categorized as having either a good (n=68) or poor (n=17) short-term prognosis. Notably, there were no statistically significant differences in RC and APOC3 levels between the two categories (**Figure 4B**).

### Serum lipids in GBS with or without cranial nerve damage or limb pain

**Figure 5:**
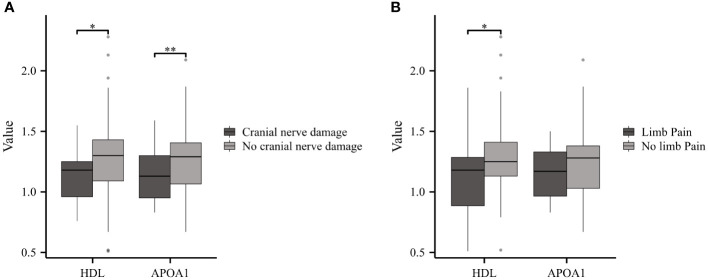
Serum Lipids in GBS with or without Cranial Nerve Damage or Limb Pain. **(A)** GBS patients with cranial nerve damage displayed reduced levels of HDL and APOA1 compared to those No cranial nerve damage. **(B)** GBS patients experiencing limb pain showed notably lower HDL levels compared to the No limb pain group (*p<0.05, **p<0.01).

In this cohort, GBS patients presenting symptoms of cranial nerve damage or limb pain were classified as the experimental group, while the remaining patients formed the control group. GBS patients with cranial nerve damage (n=25) exhibited reduced levels of HDL and APOA1 compared to those without damage (n=59) (**Figure 5A**). Furthermore, GBS patients with limb pain (n=23) showed significantly lower HDL levels in comparison to the control group (n=61) (**Figure 5B**).

### Serum lipids show associations with inflammatory markers in GBS

**Figure 6:**
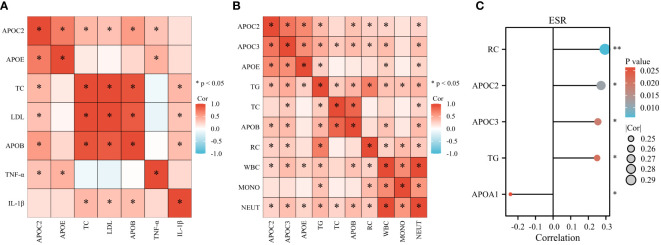
Serum Lipids show associations with Inflammatory Markers in GBS. **(A)** Positive correlations were observed between APOC2 and APOE with tumor necrosis factor -α (TNF-α). TC, APOB, and LDL exhibited positive associations with Interleukin 1β (IL-1β). **(B)** Multiple lipids were found to be positively associated with inflammatory cells: APOC2, APOC3, APOE, TG, APOB, and RC with white blood cells (WBC); TG and RC with monocytes (MONO); APOC2, APOC3, APOE, TG, TC, APOB, and RC with neutrophils (NEUT). **(C)** Positive associations were observed between APOC2, APOC3, TG, and RC with erythrocyte sedimentation rate (ESR). A negative correlation was found between APOA1 and ESR (*p<0.05).

The present study investigated various inflammatory markers, including cytokines (TNF-α, IL-1β), white blood cells (WBC), monocytes (MONO), neutrophils (NEUT), and erythrocyte sedimentation rate (ESR). The results revealed positive correlations between APOC2 and APOE with TNF-α. Additionally, TC, APOB, and LDL exhibited positive associations with IL-1β (**Figure 6A**, n=42). In **Figure 6B** (n=85), multiple lipids were found to be positively associated with inflammatory cells (APOC2, APOC3, APOE, TG, APOB, and RC with WBC; TG and RC with MONO; APOC2, APOC3, APOE, TG, TC, APOB, and RC with NEUT). Moreover, we observed positive associations between APOC2, APOC3, TG, and RC with ESR; however, a negative correlation was found between APOA1 and ESR (**Figure 6C**, n=85).

### Serum lipids were found to be related to immune status of GBS

**Figure 7:**
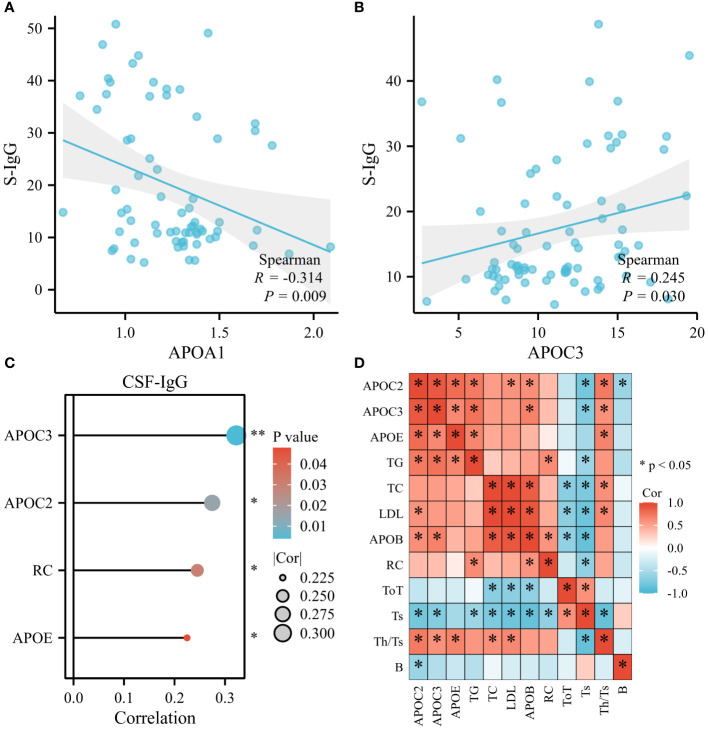
Serum Lipids were found to be related to Immune Status of GBS. **(A)** APOA1 displayed a remarkable inverse correlation with serum IgG (S-IgG). **(B)** Positive associations were observed between APOC3 and S-IgG. **(C)** APOC2, APOC3, APOE, and RC demonstrated prominent and positive correlations with CSF-IgG. **(D)** Most lipids exhibited negative associations with lymphocyte subsets: APOB, LDL, and TC with CD3+ T cells (ToT); APOC2, APOC3, TG, TC, LDL, APOB, and RC with CD8+ T cells (Ts); APOC2 with CD19+ cells (B cells). However, substantially positive correlations were noted between APOC3, APOE, TC, LDL, and CD4+/CD8+ ratio (Th/Ts) (*p<0.05, **p<0.01).

Certain indices can indirectly reflect the immune status of the body, such as immunoglobulins (IgG, IgA, IgM) and lymphocyte subsets, including CD19^+^ cells (B cells), CD3^+^ T cells (ToT), CD4^+^ T cells (Th), CD8^+^ T cells (Ts), and the CD4^+^/CD8^+^ ratio (Th/Ts). As shown in **Figure 7A** (n=70), APOA1 displayed a significant inverse correlation with serum IgG (S-IgG), while positive associations were observed between APOC3 and S-IgG (**Figure 7B**, n=78). Furthermore, APOC2, APOC3, APOE, and RC demonstrated significant and positive correlations with CSF-IgG (**Figure 7C**, n=78). Most lipids exhibited negative associations with lymphocyte subsets (APOB, LDL, and TC with ToT; APOC2, APOC3, TG, TC, LDL, APOB, and RC with Ts cells; APOC2 with B cells). However, significantly positive correlations were noted between APOC3, APOE, TC, LDL, and Th/Ts (**Figure 7D**, n=12).

### Serum lipids with immunotherapy

**Figure 8:**
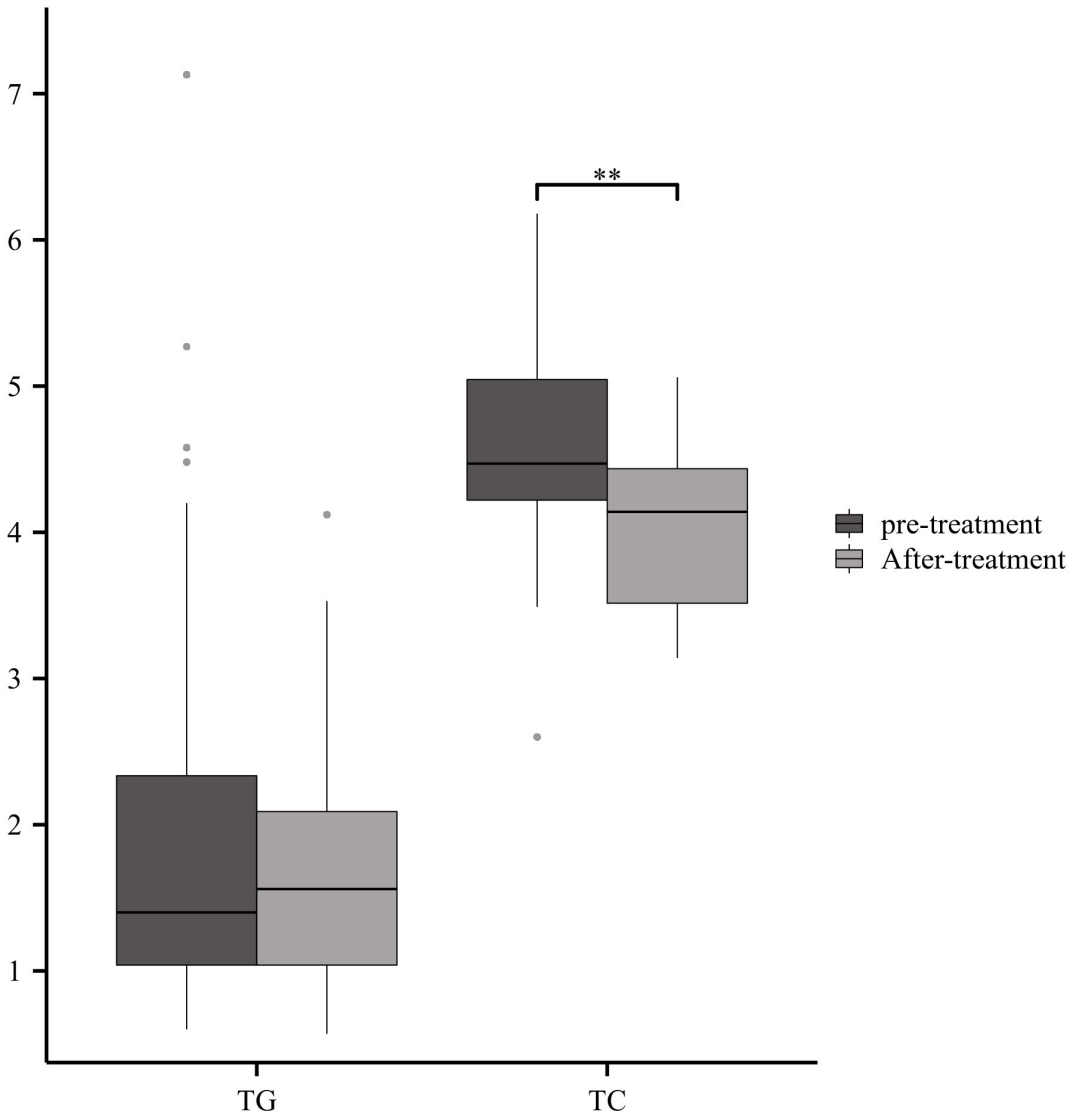
Serum Lipids with immunotherapyGBS patients showed a significant reduction in TC after intravenous immunoglobulin therapy (**p<0.01).

To date, intravenous immunoglobulin and plasma exchange are the only recognized immunotherapeutic drugs that can accelerate recovery in GBS. We retrospectively analyzed the changes in serum lipids before and after treatment in 27 patients with GBS, which showed a significant reduction in TC after intravenous immunoglobulin therapy (**Figure 8**).

## Discussion

To the best of our knowledge, this study represents the first comprehensive investigation of the association between serum lipids and GBS. Our findings unveiled elevated levels of APOC2, APOC3, APOE, TG, APOB, and RC in GBS patients compared to healthy controls, whereas APOA2, APOA1, and HDL were lower. No statistically significant differences were observed in TC, LDL, oxLDL, and LPa between the two groups. These outcomes are consistent with a study by Bin Zhang et al., who reported decreased expression of APOA1 in GBS and other autoimmune demyelinating diseases (20). Furthermore, previous research also supports the increased levels of TG, TC, HDL, LDL, RC, APOA1, and APOB in GBS patients (21). However, this study is the first to report the detection of APOA2, APOC2, oxLDL, and LPa in GBS and their comparison with healthy controls.

The finding of higher levels of serum ApoE in GBS patients compared to healthy controls, especially when contrasted with decreased levels of ApoE in the cerebrospinal fluid (CSF), suggests an intriguing and complex relationship between ApoE and GBS (9). ApoE is produced in different tissues, including the liver and the central nervous system. The source of ApoE in serum and CSF is different. Serum ApoE primarily comes from the liver, while CSF ApoE may have contributions from the brain and other neural tissues. Variations in ApoE levels between these compartments can be due to differences in local production and metabolism. GBS is characterized by an immune-mediated inflammatory response, particularly in the peripheral nervous system. Elevated serum ApoE levels could be a part of the systemic immune response to inflammation and tissue damage. ApoE has been associated with anti-inflammatory and immunomodulatory properties. In the context of GBS, the peripheral nerves are the primary target of the autoimmune attack. The higher serum ApoE levels might reflect a systemic response to the tissue damage and potential repair processes occurring in peripheral nerves (22, 23). ApoE is known to play a role in neuronal repair and regeneration. The contrasting levels of ApoE in serum and CSF could have biomarker potential. Measuring ApoE levels in both compartments may provide valuable information for diagnosing and monitoring GBS and potentially predicting its course and outcomes. In summary, the higher levels of serum ApoE in GBS patients suggest that ApoE is involved in the immune response and potential repair processes related to the peripheral nervous system damage in GBS. The difference between serum and CSF levels likely reflects the complex biology of ApoE in different tissues and its role in immune regulation and neural repair. Further research is needed to fully understand the implications of these findings and their potential clinical significance in GBS.

In a previous study, significantly higher levels of APOC3 were detected in the CSF and serum of GBS patients compared to other neurological diseases and healthy control groups (5). Similarly, current analysis revealed elevated expression of APOC3 in severe GBS cases and GBS patients with positive CSF neurovirus IgG. Numerous studies have demonstrated that APOC3 can activate alternative inflammasomes, triggering inflammation and organ damage (6, 24, 25). Moreover, experimental animal studies have shown that APOC3 can exacerbate tissue damage and delay repair (6). If APOC3 is found to be a key factor in GBS-related inflammation, it could open up possibilities for developing targeted therapies aimed at modulating APOC3 or its effects, potentially reducing the severity of inflammation and the progression of GBS. The identification of APOC3 as a factor associated with GBS-related inflammation might lead to the development of diagnostic and prognostic biomarkers. Detecting elevated levels of APOC3 or other associated molecules could help in the early diagnosis and management of GBS. Understanding the role of APOC3 in GBS-related inflammation could shed light on the underlying mechanisms of the disorder. It might provide insights into how the immune system responds to peripheral nerve damage and whether APOC3 plays a role in this response.

In this study, we observed that severe GBS cases exhibited high levels of TC, while GBS patients who required re-admission showed elevated levels of RC. These findings are in line with previous discovery that RC is a risk factor for both GBS and severe GBS (21). The research conducted by Zhao Jia Jun et al. introduced the concept of “cholesterol toxicity<city/>,” which proposes that elevated cholesterol levels are a contributing factor to several common chronic conditions, including liver diseases, diabetes, chronic kidney disease, Alzheimer’s disease, osteoporosis, osteoarthritis, and pituitary-thyroid axis dysfunction (12). Additionally, excessive cholesterol accumulation has been linked to immune dysfunction (12). Oxysterols, including 27-hydroxycholesterol, are oxidized forms of cholesterol. They have been shown to influence inflammatory processes. 27-hydroxycholesterol, in particular, has been linked to the upregulation of inflammatory cytokines, indicating its potential role in promoting inflammation. Oxysterols, including 27-hydroxycholesterol, can influence the behavior of macrophages, which play a central role in both cholesterol metabolism and the immune response. Oxysterols can promote pro-inflammatory phenotypes in macrophages. Oxysterols and immune system dysregulation can promote inflammation and impact immune responses and that from the findings in this paper, we speculate that inflammation has a key role to play in nerve damage. GBS is considered an autoimmune disorder, and factors that influence immune responses and self-recognition may play a role in its development. Dysregulation in immune responses, including the Th1/Th2 balance, can potentially influence autoimmunity. Cholesterol is a component of myelin, the protective sheath around nerve fibers. Altered cholesterol metabolism may affect myelin integrity, which, in some forms of GBS, is targeted by the immune system. Moreover, 27-hydroxycholesterol induces an upregulation of inflammatory cytokines, such as IL-8, IL-1β, and TNF-α, in monocytes via the TLR4/NF-κB pathway (26). Statins, widely prescribed lipid-lowering drugs, have demonstrated beneficial therapeutic effects in animal models of GBS (13). However, the precise role of cholesterol in the pathogenesis of GBS and its association with severe GBS remains to be fully elucidated.

Compared with healthy individuals, patients with GBS exhibited decreased levels of APOA1 and HDL. Moreover, patients experiencing cranial nerve damage or limb pain demonstrated reduced expression of these two lipids. This observation aligns with the notion that APOA1 and HDL function as protective lipids (27–29). APOA1 is a characteristic apolipoprotein found in HDL particles. HDL facilitates the efflux of cholesterol from cells into the peripheral circulation, possesses antioxidant enzymes such as paraoxonase, and exerts anti-inflammatory properties through the inhibition of adhesion molecule expression on endothelial cell membranes and monocyte recruitment inhibition (30). HDL is known for its anti-inflammatory properties. It can help reduce inflammation in blood vessels and limit damage caused by inflammation. While GBS is not primarily an inflammatory disorder, inflammation can be a secondary effect, and the anti-inflammatory properties of HDL may have a modulating influence. HDL plays a key role in transporting cholesterol away from peripheral tissues and back to the liver for metabolism. In some forms of GBS, such as the Miller Fisher variant, the immune system may target nerve structures containing cholesterol-rich components like gangliosides. HDL may have neuroprotective properties that could indirectly affect the course and outcomes of GBS. Protecting nerve structures from oxidative damage or inflammation may be relevant in the context of neurological disorders.

Additionally, this study presents novel findings indicating differential expression of oxLDL among different GBS subtypes. GBS patients with pre-infection exhibited higher levels of TG, TC, and HDL compared to controls. However, further investigation is necessary to decipher the exact underlying causes of these observations.

TNF-α and IL-1β, prominent pro-inflammatory cytokines, are secreted by Th1 cells. Accumulating evidence indicates that these cytokines play crucial roles in the initiation and progression of GBS and EAN (31–33). Several studies have reported significantly elevated levels of ESR in GBS patients when compared to healthy individuals (34, 35). Moreover, numerous studies have demonstrated higher ratios of neutrophil-to-lymphocyte (NLR) and monocyte-to-lymphocyte ratio (MLR) in GBS patients than in healthy controls. When comparing the mild and severe groups, the severe group exhibited even higher NLR and MLR (36, 37). Jahan et al. proposed a positive correlation between monocyte count and the severity of GBS (36). Ren et al. discovered that patients with acute-onset GBS showed significantly increased neutrophil ratios and counts compared to healthy controls. These counts/ratios decreased during remission but rose again in patients with recurrent GBS (38). Our findings demonstrate a positive association between serum lipids and inflammatory markers, including TNF-α, IL-1β, ESR, WBC, monocytes, and neutrophils in GBS patients. Only APOA1 displayed a negative correlation with ESR. These data suggest that lipids are strongly associated with inflammatory markers in patients with GBS.

The immune status can be assessed by evaluating humoral immunity (including immunoglobulins G, A, and M) and cellular immunity, which encompasses CD19^+^ B cells, CD3^+^ T cells, CD4^+^ T cells, CD8^+^ T cells, and the CD4^+^/CD8^+^ ratio. Previous research has demonstrated that individuals with GBS exhibit elevated proportions of CD4^+^ T cells and the CD4^+^/CD8^+^ T cell ratio in CSF, while CD8^+^ T cells are decreased in individuals with GBS (39). Furthermore, some scholars have indicated that alterations in T lymphocyte subsets, particularly CD4^+^ T cells, may play a significant role in the pathogenesis of AIDP and the mechanism of action of intravenous immunoglobulin (IVIG) against AIDP (40). GBS patients exhibited notable reductions in the percentage of CD3^+^ T cells compared with individuals with other neuropathies, as well as a decrease in the proportion of CD8^+^ T cells compared with healthy controls (41). Currently, IVIG is a commonly used and relatively safe treatment for GBS (42). After IVIG treatment, there was an increase in the CD4^+^/CD8^+^ T cell ratio, while the percentage of CD8^+^ T cells and CD19^+^ B cells decreased significantly (40). Research has demonstrated that the concentrations of IgG, IgA, and IgM during the acute phase of GBS were significantly higher than those in normal controls (43, 44). Our findings revealed a significant negative correlation between APOA1 and serum IgG levels, whereas APOC3 displayed a positive correlation with serum IgG levels. Additionally, APOC2, APOC3, APOE, and RC were significantly and positively associated with CSF IgG levels. APOB, LDL, and TC exhibited negative correlations with CD3^+^ T cells, while APOC2, APOC3, TG, TC, LDL, APOB, and RC showed negative correlations with CD8^+^ T cells. APOC2 exhibited a negative correlation with CD19^+^ B cells. Noteworthy positive associations were observed among APOC3, APOE, TC, LDL, and the CD4^+^/CD8^+^ T cell ratio. Furthermore, previous studies have proposed that APOA1 functions as an immune regulator, capable of suppressing pro-inflammatory cytokines produced by activated T cells in specific autoimmune diseases (20). Silvia Iannello demonstrated that low TG levels may serve as an early marker of autoimmunity or hyperactivity of the immune system (45). Taken collectively, lipids are closely related to the immune status of GBS.

## Conclusion

This research comprehensively analyzed the serum lipids profile of GBS. The findings exhibit heightened expression levels of APOC2, APOC3, APOE, TG, APOB, and RC in GBS subjects, while protective APOA2, APOA1, and HDL were observed as downregulated. Moreover, lipids were closely associated with the severity, subtypes, clinical outcomes, precursor infections, clinical symptoms, immunotherapy, inflammation, and immune status of GBS. This suggests that low-fat diets or lipid-lowering drugs may be useful for the treatment of GBS, providing a new perspective for the clinical treatment of GBS.

## Data availability statement

The original contributions presented in the study are included in the article/supplementary material. Further inquiries can be directed to the corresponding author.

## Ethics statement

The studies involving humans were approved by the institutional review boards of Beijing Tiantan Hospital (batch number: KY-2022-039-01). The studies were conducted in accordance with the local legislation and institutional requirements. Written informed consent for participation in this study was provided by the participants’ legal guardians/next of kin. Written informed consent was obtained from the individual(s), and minor(s)’ legal guardian/next of kin, for the publication of any potentially identifiable images or data included in this article.

## Author contributions

LW: Writing – review & editing, Writing – original draft. YD: Writing – review & editing. JL: Writing – review & editing, Resources. GHZ: Writing – review & editing, Methodology. SL: Writing – review & editing, Resources. WJ: Writing – review & editing, Investigation. KC: Writing – review & editing, Supervision. XL: Writing – review & editing, Data curation. YC: Writing – review & editing, Formal analysis. SW: Writing – review & editing, Validation. GJZ: Writing – review & editing, Project administration, Supervision.

## References

[1] Willison HJ Jacobs BC van Doorn PA. Guillain-Barré syndrome. Lancet (2016) 388(10045):717–27. doi: 10.1016/S0140-6736(16)00339-1 26948435

[2] Esposito S Longo MR. Guillain-Barré syndrome. Autoimmun Rev (2017) 16(1):96–101. doi: 10.1016/j.autrev.2016.09.022 27666816

[3] Lu MO Zhu J. The role of cytokines in Guillain-Barré syndrome. J Neurol (2011) 258(4):533–48. doi: 10.1007/s00415-010-5836-5 21104265

[4] Hughes RA Cornblath DR. Guillain-Barré syndrome. Lancet (2005) 366(9497):1653–66. doi: 10.1016/S0140-6736(05)67665-9 16271648

[5] Ding Y Shi Y Wang L Li G Osman RA Sun J. Potential biomarkers identified by tandem mass tags based quantitative proteomics for diagnosis and classification of Guillain-Barre syndrome. Eur J Neurol (2022) 29(4):1155–64. doi: 10.1111/ene.15213 34913222

[6] Zewinger S Reiser J Jankowski V Alansary D Hahm E Triem S. Apolipoprotein C3 induces inflammation and organ damage by alternative inflammasome activation. Nat Immunol (2020) 21(1):30–41. doi: 10.1038/s41590-019-0548-1 31819254

[7] Zhang HL Wu J Zhu J. The role of apolipoprotein E in Guillain-Barre syndrome and experimental autoimmune neuritis. J BioMed Biotechnol 2010. (2010) p:357412. doi: 10.1155/2010/357412 PMC2825561 20182542

[8] Zhang HL Mao XJ Zhang XM Li HF Zheng XY Adem A. APOE epsilon3 attenuates experimental autoimmune neuritis by modulating T cell, macrophage and Schwann cell functions. Exp Neurol (2011) 230(2):197–206. doi: 10.1016/j.expneurol.2011.04.016 21550340

[9] Yu S Duan RS Chen Z Quezada HC Bao L Nennesmo I. Increased susceptibility to experimental autoimmune neuritis after upregulation of the autoreactive T cell response to peripheral myelin antigen in apolipoprotein E-deficient mice. J Neuropathol Exp Neurol (2004) 63(2):120–8. doi: 10.1093/jnen/63.2.120 14989598

[10] Georgila K Vyrla D Drakos E Apolipoprotein A-I. (ApoA-I), immunity, inflammation and cancer. Cancers (Basel) (2019) 11(8):1097. doi: 10.3390/cancers11081097 31374929 PMC6721368

[11] Kober AC Manavalan APC Tam-Amersdorfer C Holmér A Saeed A Fanaee-Danesh E. Implications of cerebrovascular ATP-binding cassette transporter G1 (ABCG1) and apolipoprotein M in cholesterol transport at the blood-brain barrier. Biochim Biophys Acta Mol Cell Biol Lipids (2017) 1862(6):573–88. doi: 10.1016/j.bbalip.2017.03.003 28315462

[12] Song Y Liu J Zhao K Gao L Zhao J. Cholesterol-induced toxicity: An integrated view of the role of cholesterol in multiple diseases. Cell Metab (2021) 33(10):1911–25. doi: 10.1016/j.cmet.2021.09.001 34562355

[13] Langert KA Goshu B Stubbs EB Jr. Attenuation of experimental autoimmune neuritis with locally administered lovastatin-encapsulating poly(lactic-co-glycolic) acid nanoparticles. J Neurochem (2017) 140(2):334–46. doi: 10.1111/jnc.13892 PMC5225029 27861905

[14] Reindl M Knipping G Wicher I Dilitz E Egg R Deisenhammer F. Increased intrathecal production of apolipoprotein D in multiple sclerosis. J Neuroimmunol (2001) 119(2):327–32. doi: 10.1016/S0165-5728(01)00378-2 11585636

[15] Asbury AK Cornblath DR. Assessment of current diagnostic criteria for Guillain-Barre syndrome. Ann Neurol (1990) 27 Suppl:S21–4. doi: 10.1002/ana.410270707 2194422

[16] Hughes RA Newsom-Davis JM Perkin GD Pierce JM. Controlled trial prednisolone in acute polyneuropathy. Lancet (1978) 2(8093):750–3. doi: 10.1016/S0140-6736(78)92644-2 80682

[17] Uncini A Manzoli C Notturno F Capasso M. Pitfalls in electrodiagnosis of Guillain-Barré syndrome subtypes. J Neurol Neurosurg Psychiatry (2010) 81(10):1157–63. doi: 10.1136/jnnp.2010.208538 20870864

[18] Ho TW Mishu B Li CY Gao CY Cornblath DR Griffin JW. Guillain-Barré syndrome in northern China. Relationship to Campylobacter jejuni infection and anti-glycolipid antibodies. Brain (1995) 118(Pt 3):597–605. doi: 10.1093/brain/118.3.597 7600081

[19] Jacobs BC Rothbarth PH van der Meché FG Herbrink P Schmitz PI de Klerk MA. The spectrum of antecedent infections in Guillain-Barré syndrome: a case-control study. Neurology (1998) 51(4):1110–5. doi: 10.1212/WNL.51.4.1110 9781538

[20] Zhang B Pu S Li B Ying J Song XW Gao C. Comparison of serum apolipoprotein A-I between Chinese multiple sclerosis and other related autoimmune disease. Lipids Health Dis (2010) 9:34. doi: 10.1186/1476-511X-9-34 20350318 PMC2860353

[21] Ding Y Wang L Sun J Shi Y Li G Luan X. Remnant cholesterol and dyslipidemia are risk factors for Guillain-Barre syndrome and severe Guillain-Barre syndrome by promoting monocyte activation. Front Immunol (2022) 13:946825. doi: 10.3389/fimmu.2022.946825 35911688 PMC9326451

[22] Jin T Hu LS Chang M Wu J Winblad B Zhu J. Proteomic identification of potential protein markers in cerebrospinal fluid of GBS patients. Eur J Neurol (2007) 14(5):563–8. doi: 10.1111/j.1468-1331.2007.01761.x 17437617

[23] Gaillard O Gervais A Meillet D Plassart E Fontaine B Lyon-Caen O. Apolipoprotein E and multiple sclerosis: a biochemical and genetic investigation. J Neurol Sci (1998) 158(2):180–6. doi: 10.1016/S0022-510X(98)00118-X 9702689

[24] Hsu CC Shao B Kanter JE He Y Vaisar T Witztum JL. Apolipoprotein C3 induces inflammasome activation only in its delipidated form. Nat Immunol (2023) 24(3):408–11. doi: 10.1038/s41590-023-01423-2 PMC9992333 36781985

[25] Gong T Zhou R. ApoC3: an 'alarmin' triggering sterile inflammation. Nat Immunol (2020) 21(1):9–11. doi: 10.1038/s41590-019-0562-3 31822868

[26] Gargiulo S Gamba P Testa G Rossin D Biasi F Poli G. Relation between TLR4/NF-kappaB signaling pathway activation by 27-hydroxycholesterol and 4-hydroxynonenal, and atherosclerotic plaque instability. Aging Cell (2015) 14(4):569–81. doi: 10.1111/acel.12322 PMC4531071 25757594

[27] Lecamwasam A Mansell T Ekinci EI Saffery R Dwyer KM. Blood plasma metabolites in diabetes-associated chronic kidney disease: A focus on lipid profiles and cardiovascular risk. Front Nutr (2022) 9:821209. doi: 10.3389/fnut.2022.821209 35295919 PMC8918794

[28] Heier M Ofstad AP Borja MS Brunborg C Endresen K Gullestad L. High-density lipoprotein function is associated with atherosclerotic burden and cardiovascular outcomes in type 2 diabetes. Atherosclerosis (2019) 282:183–7. doi: 10.1016/j.atherosclerosis.2018.07.005 30017177

[29] Cochran BJ Ong KL Manandhar B Rye KA. APOA1: a protein with multiple therapeutic functions. Curr Atheroscler Rep (2021) 23(3):11. doi: 10.1007/s11883-021-00906-7 33591433

[30] Lorincz B Jury EC Vrablik M Ramanathan M Uher T. The role of cholesterol metabolism in multiple sclerosis: From molecular pathophysiology to radiological and clinical disease activity. Autoimmun Rev (2022) 21(6):103088. doi: 10.1016/j.autrev.2022.103088 35398271

[31] Sun T Chen X Shi S Liu Q Cheng Y. Peripheral blood and cerebrospinal fluid cytokine levels in Guillain Barre syndrome: A systematic review and meta-analysis. Front Neurosci (2019) 13:717. doi: 10.3389/fnins.2019.00717 31379477 PMC6646663

[32] Matsui H Ohgomori T Natori T Miyamoto K Kusunoki S Sakamoto K. Keratan sulfate expression in microglia is diminished in the spinal cord in experimental autoimmune neuritis. Cell Death Dis (2013) 4(12):e946. doi: 10.1038/cddis.2013.479 24309933 PMC3877550

[33] Du Y Zhang G Zhang Z Wang Q Ma R Zhang L. Toll-like receptor 2 and -4 are involved in the pathogenesis of the Guillain-Barre syndrome. Mol Med Rep (2015) 12(2):3207–13. doi: 10.3892/mmr.2015.3730 25954926

[34] Sunmonu TA Komolafe MA Adewuya A Olugbodi AA. Clinically diagnosed Guillain-Barre syndrome in Ile-Ife, Nigeria. West Afr J Med (2008) 27(3):167–70. 19256323

[35] Li X Li W Shi X Mo L Luo Y Qin L. Is serum bilirubin associated with the severity of Guillain-Barre syndrome? Int J Neurosci (2018) 128(5):595–9. doi: 10.1080/00207454.2018.1431005 29130362

[36] Jahan I Ahmed R Ahmed J Khurshid S Biswas PP Upama IJ. Neutrophil-lymphocyte ratio in Guillain-Barré syndrome: A prognostic biomarker of severe disease and mechanical ventilation in Bangladesh. J Peripher Nerv Syst (2023) 28(1):47–57. doi: 10.1111/jns.12531 36700342 PMC10155239

[37] Xu L Gao TX Chang SH Jiang SM Zhang LJ Yang L. Role of lymphocyte-related immune-inflammatory biomarkers in detecting early progression of Guillain-Barre syndrome. J Clin Neurosci (2022) 105:31–6. doi: 10.1016/j.jocn.2022.08.017 36063751

[38] Ren K Yang A Lu J Zhao D Bai M Ding J. Association between serum low-density neutrophils and acute-onset and recurrent Guillain-Barre syndrome. Brain Behav (2022) 12(1):e2456. doi: 10.1002/brb3.2456 34894104 PMC8785626

[39] Cao L. [Preliminary studies of T lymphocyte subsets in patients with neurologic diseases]. Zhonghua Shen Jing Jing Shen Ke Za Zhi (1990) 23(3):159–61, 190. 2143977

[40] Hou HQ Miao J Feng XD Han M Song XJ Guo L. Changes in lymphocyte subsets in patients with Guillain-Barre syndrome treated with immunoglobulin. BMC Neurol (2014) 14:202. doi: 10.1186/s12883-014-0202-3 25315010 PMC4210538

[41] Harness J McCombe PA. Increased levels of activated T-cells and reduced levels of CD4/CD25+ cells in peripheral blood of Guillain-Barre syndrome patients compared to controls. J Clin Neurosci (2008) 15(9):1031–5. doi: 10.1016/j.jocn.2007.09.016 18653346

[42] Rajabally YA. Immunoglobulin and monoclonal antibody therapies in Guillain-Barre syndrome. Neurotherapeutics (2022) 19(3):885–96. doi: 10.1007/s13311-022-01253-4 PMC9159039 35648286

[43] Dowling PC Bosch VV Cook SD Chmel H. Serum immunoglobulins in Guillain-Barre syndrome. J Neurol Sci (1982) 57(2-3):435–40. doi: 10.1016/0022-510X(82)90047-8 7161628

[44] Araga S Kagimoto H Adachi A Funamoto K Inoue K Takahashi K. Kappa/lambda ratios of IgG, IgA and IgM in the cerebrospinal fluid of patients with Guillain-Barre syndrome. Jpn J Med (1991) 30(2):118–22. doi: 10.2169/internalmedicine1962.30.118 1907691

[45] Iannello S Cavaleri A Milazzo P Cantarella S Belfiore F. Low fasting serum triglyceride level as a precocious marker of autoimmune disorders. MedGenMed (2003) 5(3):20. 14600656

